# Light transmittance and surface roughness of a feldspathic ceramic CAD-CAM material as a function of different surface treatments

**DOI:** 10.1186/s12903-016-0245-5

**Published:** 2016-07-15

**Authors:** Çağrı Ural, İbrahim Duran, Betül Evmek, İdris Kavut, Seda Cengiz, Emir Yuzbasioglu

**Affiliations:** Department of Prosthodontics, Faculty of Dentistry, Ondokuz Mayıs University, Samsun, Turkey; Department of Prosthodontics, Faculty of Dentistry, Bülent Ecevit University, Zonguldak, Turkey; Department of Prosthodontics, School of Dentistry, Istanbul Medipol University, Ataturk Bulvari, No:27, 34083 Unkapani, Fatih Istanbul, Turkey; Biomaterials and Translational Dental Research Laboratory, Regenerative and Restorative Medicine Research Center (REMER), Istanbul Medipol University, Istanbul, Turkey

**Keywords:** CAD-CAM blocks, Feldspathic ceramic, Light-transmission, Laser treatment

## Abstract

**Background:**

The aim of the present study was to determine the effect of different surface treatments on light transmission of aesthetic feldspathic ceramics used in CAD-CAM chairside restorations.

**Methods:**

Forty eight feldspatic ceramic test specimens were prepared from prefabricated CAD-CAM blocks by using a slow speed diamond saw. Test specimens were prepared and divided into 4 groups (*n* = 12). In the control group, no surface treatments were applied on the feldspathic ceramic surfaces. In the hydrofluoric acid group, the bonding surfaces of feldspathic ceramics were etched with 9.5 % hydrofluoric acid. In the sandblasting group the feldspathic ceramic surfaces were air-abraded with 30-μm alumium oxide (Al_2_O_3_) particles and Er:YAG laser was used to irradiate the ceramic surfaces. The incident light power given by the LED device and the transmitted light power through each ceramic sample was registered using a digital LED radiometer device. Each polymerization light had a light guide with 8-mm-diameter tips. Light transmission of feldspathic ceramic samples was determined by placing it on the radiometer and irradiating the specimen for 10 s at the highest setting for each light polymerization. All specimens were coated with gold using a sputter coater and examined under a field emission scanning electron microscope. Surface roughness measurement each group were evaluated with 3D optical surface and tactile profilometers.

**Results:**

One-way ANOVA test results revealed that both surface conditioning method significantly affect the light transmittance (F:412.437; *p* < 0.001) and the surface roughness values (F:16.386; *p* < 0.001). Al_2_O_3_ and Er-YAG laser application reduced the light transmission significantly (*p* < 0.05).

**Conclusions:**

The laser and Al_2_O_3_ applications reduced the light transmission of 1.5 mm thickness feldspathic ceramic material below the value of 400 mW/cm^2^ which is critical limit for safe polymerization.

## Background

The all ceramic materials and tooth-colored restorative materials have been widely used in dental practice to meet patients’ aesthetic demands [1]. Ceramic veneers are the most common choice for achieving aesthetics in anterior region and they can be fabricated from various ceramics [1]. Feldspathic ceramics can be used for aesthetic chairside restoration and which is biocompatible and mimic the natural tooth enamel like shading, strength and abrasion resistance [2]. These type of restorations must be cemented to the natural tooth structure with an adhesive resin cement. There are some studies suggested, acid etching and airborne particle abrasion to enhance the bonding by modifying the ceramic surface that optimizes the micromechanical retention [3, 4]. In recent years another technique that frequently used for conditioning of ceramic surfaces by laser irradiation. Er:YAG laser is most recommended type of laser to be used due to its good interaction with dental tissues and proper choice for repair of ceramic materials [5]. According to Gökçe et al. [6] Er-YAG laser application could be used for surface treatment of an aesthetic ceramic to increase bond strength.

Now it is clear that increased surface roughness with various surface treatments may increase the bonding strength of some ceramics but optical properties of ceramics such as transmittance, reflectance, and translucency of enamel and dentin porcelain are significantly influenced by surface roughness (Ra) of dentin porcelain [7]. According to a recent study after the application of the surface treatments the color change of the ceramics were increased [8].

Either chemically or mechanically to obtain maximum performance of resin cements (light cure & dual cure), the polymerization light should pass through the porcelain material to the light-activated components of cement. Dual-curing resin systems provide a higher degree of conversion of monomers compared to light-curing resin systems. However, use of a catalyst with anterior porcelain veneers is problematic because of the potential for discoloration [9]. For a safe polymerization of resin cement light transmission should be at least 400 mw/cm^2^ [10]. Therefore determining the factors that may affect the light transmission through porcelain veneers can be critical for clinical success.

The aim of study was to investigate the effect of surface treatment on light transmission of feldspathic ceramics used in CAD-CAM chairside restorations. The null hypotheses were that light transmission of feldspathic ceramic would not be affected by surface treatments.

## Methods

### Specimen preparation

Feldspathic specimens (*N* = 48) were fabricated by slicing CAD-CAM ceramic blocks (CEREC Blocs C, S2-M, Size 14, Sirona Dental Systems GmbH, Germany) with diamond discs in a slow-speed precision cutter (Isomet; Buchler, Ltd., Lake Bluff, USA) into 12 mm × 10 mm slices of approximately 1,55 mm thickness. All the specimens were finished, polished and taken to the final thickness of 1.5 mm with waterproof abrasive papers (400 to 1200 grit), respectively (Mecatech Z34, Presi, France). During this process, the thicknesses of specimens were repeatedly checked with a micrometer (C-master; Mitutoyo, Tokyo, Japan) to ensure a final 1.5 ± 0.05 mm thickness. Then, one side of the specimens were glazed according to the manufacturers’ recommendations and cleaned in distilled water by a ultrasonic cleaner (Eurosonic Energy, Euronda SpA, Vicenza, Italy) for 10 min and dried with oil-free air for 30 s before the surface conditioning procedures. The specimens were randomly divided into 4 subgroups (*n* = 12 per group) to be conditioned with one of the following methods:

### Surface conditioning

- Group C: No surface treatment.
- Group AA: Specimens were air-abraded with 25 μm AI_2_O_3_ particles (Korox, Bego, Bremen, Germany) with a microetcher (Airsonic Mini Sandblaster, Hager & Werken, Duisburg, Germany) for 15 s from a distance of approximately 10 mm (perpendicular to the treated surface) at 2.8 bar.
- Group HF: Specimens were etched with 9.6 % hydrofluoric acid (Bisco Inc., Schaumburg, USA) for 20 s, rinsed with distilled water for 20 s and dried with oil-free air for 30 s.
- Group L: Specimens were irradiated with Er:YAG laser (Fotona, At Fidelis, Ljubljana, Slovenia) with a contact hand-piece (R 14; 1.3 mm in diameter) under water-cooling for 20 s, 1 mm perpendicular to the surface with following parameters 500 mJ; 10 W; MSP mode; 20 Hz, 37,68 J/cm2 [11].

### Light transmittance

**Fig. 1:**
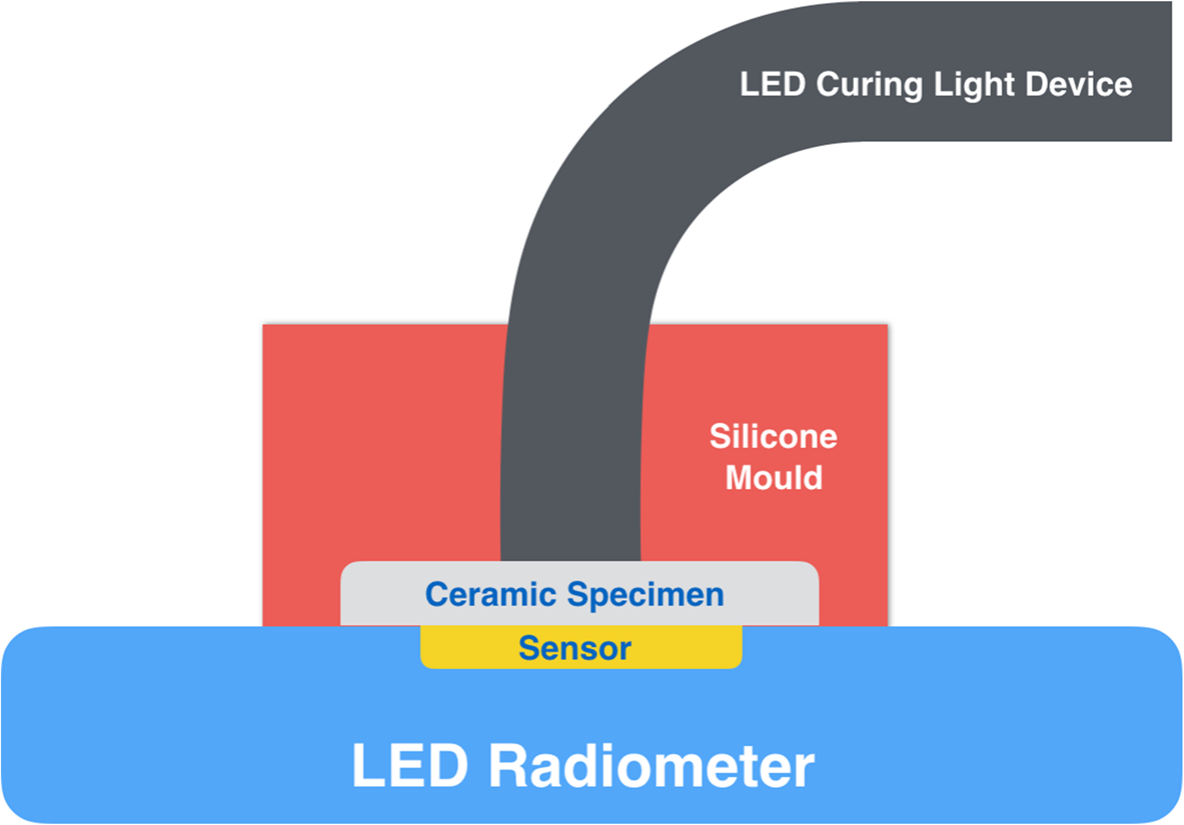
Schematic view of experimental measurement of light transmission

The incident light power given by the light emitting diode (LED) curing light device (Elipar S10, 3 M ESPE, Seefeld, Germany) and the transmitted light power through each sample was measured using a digital LED radiometer (SDI, Victoria, Australia). The LED radiometer is designed to measure the energy between 400 and 525 nm, and gives readings from 0 to 2100 mW/cm^2^. Light transmittance of specimens were measured by placing the specimen over the radiometer sensor (8 mm in diameter) and activating LED curing light device for 10 s. The tip of LED curing light device was placed in contact with glazed surface of specimen and maintained in a fixed position with a holding device (Fig. 1). During irradiation, highest and lowest values were recorded. For each sample, measurements were made in this way three times, and averages were recorded as mW/cm^2^.

### Surface roughness

Surface roughness were measured by a profilometer (Dektak 8, Veeco Ins, Plainview, NY, USA) with a 0.25-mm cutoff value, 0.01 mm resolution with the transverse length of 4.0 mm, stylus diameter of 5 μm. For determining the average roughness value, 0.5 mm/s measuring speed was used. On each specimen, six measurements were made with equal distances (1.5 mm) and the reading direction was always perpendicular to the surface of the porcelain specimens. The measured roughness parameter was Ra (arithmetical average value of all absolute distances of the roughness profile).

### Three dimesional (3D) optical profilometer

Specimens from each group was evaluated with a 3D optical surface profilometer (NanoMap-500LS, Aep Technology, Santa Clara, CA,USA) under the beam of the white light interferometry which is usually made up of the He-Ne, 633 nm [12]. The specimens were analysed by software (Digital Surf, TalyMap Platinum software, Leicester, England. Version no. 6.1.6001).

### Scanning electron microscopy (SEM)

Additional four specimens were prepared as described previously to evaluate effects of surface conditioning. Specimens were gold coated with a sputter coater (S150B; Edwards, Crawley, UK) and examined at 15 kV using a scanning electron microscope (JSM-6335 F; JEOL, Tokyo, Japan). For visual inspection, SEM photomicrographs were represented with x 500 magnification.

### Statistical analysis

The sample size was calculated using a power analysis software programme (G*Power Version 3.1.9, Dusseldorf, Germany) considering α equal to 5 %, effect size equal to 0.50 and power of 80 % according to One-Way ANAOVA test. Based on the calculations 12 specimens per group yielded to 80 % power. Data were analyzed using a statistical software programme (SPSS Software V.22, Chicago, IL, USA).

The Kolmogorov-Simirnov test showed that the data were normally distributed, One-way analysis of variance (ANOVA) and post-hoc Tukey’s HSD test were applied to analyze the data where the values of transmitted light and surface roughness were the dependent variables and surface conditioning methods (4 levels) as independent variables. *P* < 0.05 was considered to be statistically significant in all tests.

## Results

Surface conditioning methods significantly affect light transmittance (F:412.437; *p* < 0.001) and surface roughness results (F:16.386; *p* < 0.001). The descriptive statistics of light transmittance and surface roughness for each group were listed in Table 1.

**Table 1:** Mean ± standard deviation (SD), minimum (Min), maximum (Max) and 95 % Confidence Interval (CI) for mean of light transmittance (mW/cm^2^) and surface roughness (mμ) values for each group according to surface conditioning methods

Groups	Light Transmittance (mW/cm^2^)					Surface Roughness (mμ)				
	Mean ± SD	Min	Max	95 % CI for mean		Mean ± SD	Min	Max	95 % CI for mean	
				Lower Bound	Upper Bound				Lower Bound	Upper Bound
C	456.67 ± 40.58^a^	395.00	545.00	430.88	482.45	0.79 ± 0.12^a^	0.60	0.95	0.72	0.86
HF	465.83 ± 32.53^a^	430.00	525.00	445.16	486.50	1.29 ± 0.10^b^	1.06	1.42	1.23	1.36
L	399.58 ± 37.20^b^	340.00	465.00	375.95	423.22	1.13 ± 0.08^c^	1.03	1.27	1.08	1.18
AA	382.08 ± 30.71^b^	325.00	420.00	362.57	401.59	2.26 ± 0.13^d^	2.02	2.45	2.18	2.34

^a^C: Control; ^b^HF: Hydrofluoric acid; ^c^L: Laser; ^d^AA: Air-abrasion. Different upper-case letters in each column for each condition indicates significant differences (*p* < 0.05)

In experimental groups, H group (465.83 ± 32.53) presented the highest light transmittance results (mW/cm^2^) and showed no significant difference with C group. H and C groups showed significantly higher light transmittance results compared to those of other groups (L and AA) (*p* < 0.05). AA group (382.08 ± 30.71) presented the lowest light transmittance results and showed no significant difference with L group. Both AA and L surface conditioning methods significantly reduced the mean light transmittance (ranged 382.08 ± 30.71–399.58 ± 37.20) (*p* < 0.05).

Evaluated experimental groups for surface roughness, AA group (2.26 ± 0.13) presented the highest surface roughness (mμ) and showed significant difference with C, HF and L groups *p* < 0.05). C group (0.79 ± 0.12) presented the lowest surface roughness results among the tested groups. All surface conditioning methods significantly increased the mean surface roughness in all experimental groups (ranged 1.29 ± 0.10–2.26 ± 0.13) (*p* < 0.05).

**Fig. 2:**
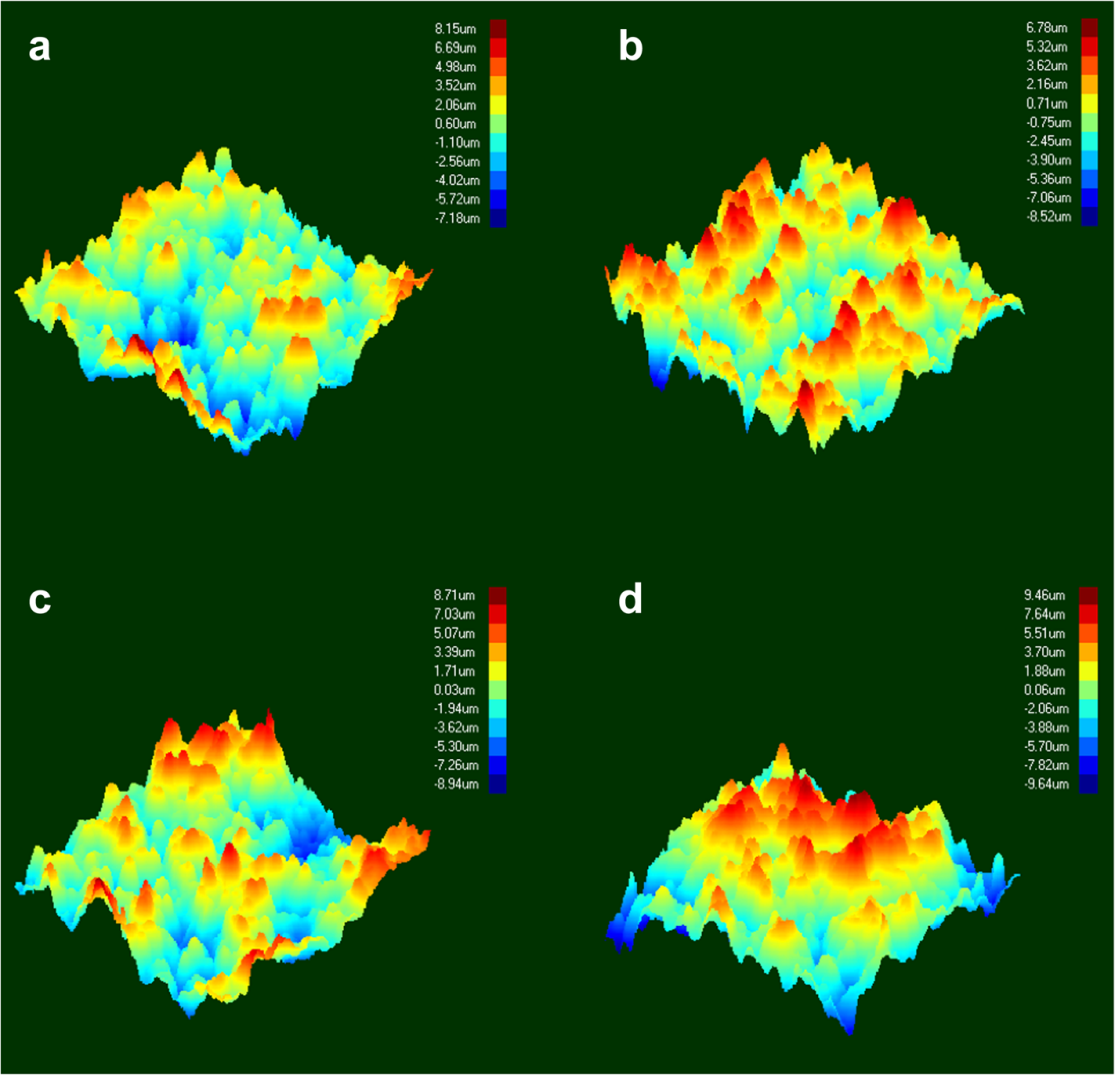
3-D optical profilometry images of feldspathic ceramic surfaces after treatment. **a** Control, **b** Laser, **c** Hydrfloric acid and **d** Air-Abrasion

**Fig. 3:**
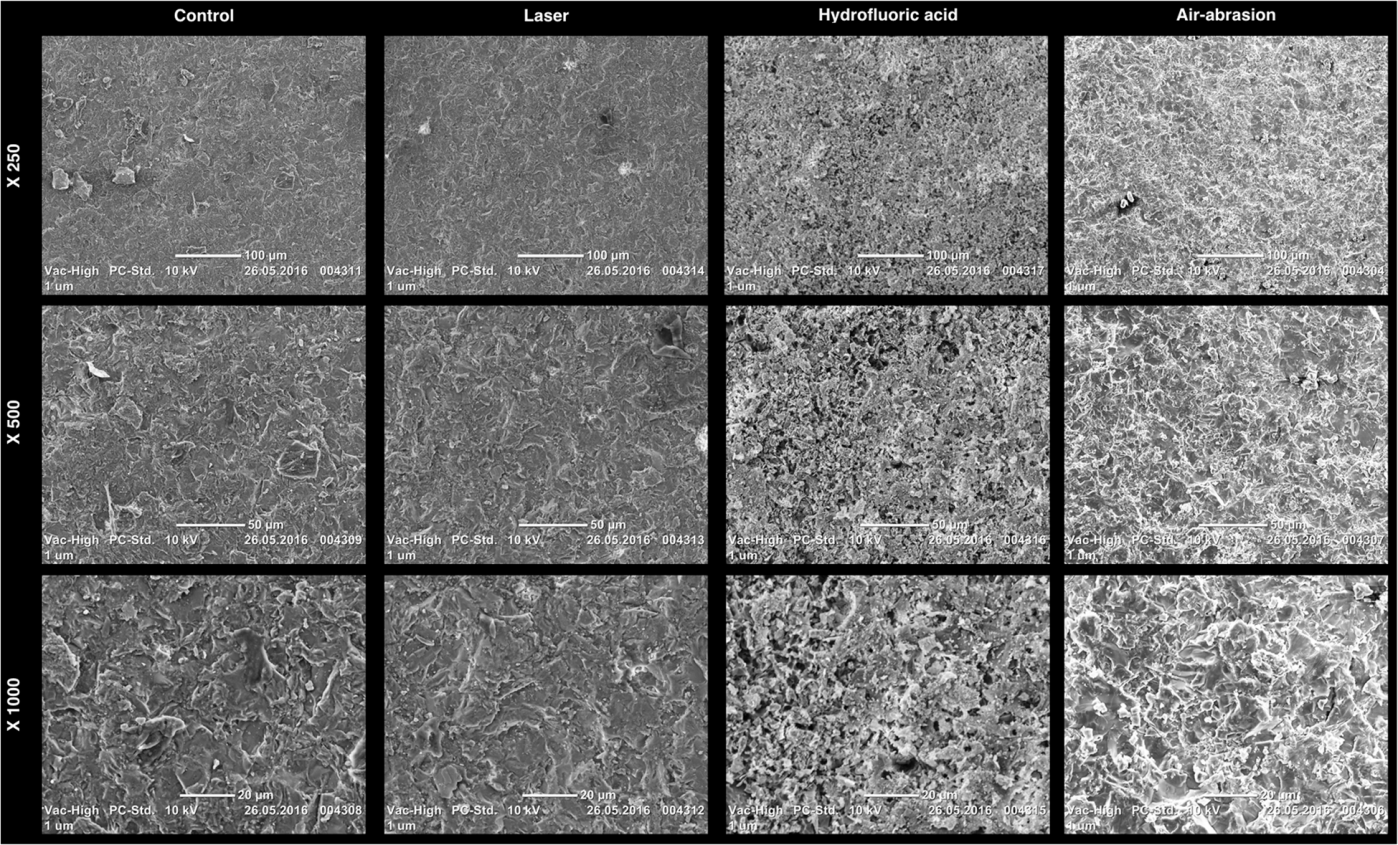
SEM micrographs of feldspathic ceramic surfaces after treatment

SEM and 3D optical profilometry images of feldspathic ceramic surfaces after treatment are presented in Figs. 2 and 3. The surface treatments showed analogical topographies, except for control group (Group C). Group C surfaces presented less irregularities with some peaks and valleys, than was achieved with other groups. Group AA had the most irregularities and sharp peaks constrated with those in other test groups.

## Discussion

Regarding to the results of this study, the null hypothesis that different surface treatment protocols on an aesthetic feldspathic ceramic system would not effect the light transmission was rejected. Although hydrofluoric acid application did not affect the light transmission, significant differences were found in light transmission after sandblasting and laser application.

Since the presentation of glass-based ceramics and discovering advantages of the adhesive cementation in dentistry, hydrofluoric acid started to be used for conditioning the restorative materials surfaces [13]. Hydrofluoric acid selectively dissolves glassy or crystalline matrix of the ceramic material and achieve a porous irregular surfaces and micro retention cites. This micro porous surfaces increases the surface area and facilitates the penetration of the resin into the ceramic surfaces. Sorenson et al. [14] stated that HF etching application onto feldspathic ceramics significantly increased the bond strength. AA is also frequently used for providing higher bond strength between resin and ceramic prior to cementation [4] and presented significantly greater mean Ra values than HF etching [15, 16]. In the present study, air abraded test specimens showed the highest surface roughness, also SEM and 3D optical profilometry images had revealed this result. More distinct sharp peaks than those of the other groups can be seen on the images.

For surface treatment, laser irradiation is frequently preferred technique for the ceramic surfaces. This technique has been proposed for surface modification and etching the ceramic inner surface [5, 17–20]. Barely, Er:YAG laser irradiation decreased bond strength, thus insufficient for feldspathic ceramic surface treatment in clinical use [5]. There are some controversial results in the literature on ceramic surface treatment by Er-YAG laser. Gökçe et al. [6] stated that 300 mJ Er-YAG could also be used for surface treatments. 500 mJ pulse energy was used for roughening the ceramic surface as performed in some recent studies [8, 21]. In this study, SEM and 3D optical profilometry images of surfaces treated with laser irradiation revealed irregular surfaces. The laser irradiated surfaces showed some irregularities with peaks and valleys, however less roughness was observed after sandblasting and hydrofluoric acid etching.

SEM images of air abraded ceramic surfaces showed more irregular and pitty surfaces compared to acid etched ceramic surfaces [22]. In recent studies it was shown that air-abraded surfaces exhibited greater surface roughness values and distinct sharper points that is compared to hydrofluoric acid etched or laser-irradiated surfaces [19, 23–25]. In this present study, surface treatments increased the surface roughness, additionally HF treatment caused micro-retentive areas and increased the roughness that is compared to sandblasting.

In the present study HF acid application, sandblasting and laser etching were performed on specimens according to manufacturer recommendations. HF etching did not effect the light transmission values of test specimens. As parallel with our findings, Turgut et al. [21] stated that HF etching didn’t yield any color change, regarding the thickness. According to them ultrasonic cleaning is the possible reason why HF etching didn’t effect optical properties of ceramics because inadequate rinsing after the etching of the porcelain surface may leave remineralized salts, and this may be caused white residue or opaque cites so this may affect the color of the ceramics [1]. Also in this in vitro research this remineralized salts can be responsible from the test results.

Sandblasted test specimens showed significant decrease in light transmission. While sandblasting, the impact of small particles on ceramic surface resulting an energy transfer, respectively. Th crystal part of the ceramic is partly absorbed this transferred energy and causing surface melting within a microscopic range [16]. Thereby the aluminum oxide partly gets incorporated within the applied surface, i.e. corundum sandblasted surfaces become Al_2_O_3_-contaminated [26]. Starting from this, modifying of the ceramic surfaces may also have resulted in changes in the optical properties of the feldspathic porcelain test specimens. The changes in light transmittance after the sandblasting procedures may attributed to this point.

According to Turgut et al. [21] the light transmittance characteristic is not the same at the roughened ceramic surface and unroughened one and the light can not passed through the ceramic with the same incidence and direction for both groups. Additionally the results of their study showed that although surface treatment with lasers had a significant effect on transparency, sandblasting was the most effective procedure. Similarly according to the this in vitro study results, sandblasting and laser application affected the amount of transmitted light through the feldspathic ceramic specimens which became rougher and opaque and changes in surface structure could alter the surface optical properties so reflection and absorption of light may be changed.

O’Keefe et al. [27] reported that opacity and thickness were affecting light transmission in thin ceramic veneers, so surface treatments which cause opacity can reduce the amount of transmitted light and reducing the amount of transmitted light can result in a reduction in degree of conversion. Rueggeberg et al. [10] described that the degree of conversion in a polymerization reaction is dependent on the energy delivered during light curing, characterized as the product of the light intensity and exposure time. Considering degree of conversion dual-curing systems are safer than light curing polymerization systems but the color change is problematic especially for aesthetic ceramics. Kılınç et al. [28] concluded that thickness of ceramic has more serious effect on polymerization contrast to color of ceramic.

If the thickness of ceramic is greater than 3 mm, it was adversely affected the polymerization of light cure and dual cure resin cements, therefore a 3-mm thickness was considered the critical threshold. For 1.5 mm thickness the present study results showed that the output power of control and HF acid groups were 461.67 and 471.25 mW/cm^2^ respectively. Although the samples were feldspathic ceramics which can be used for aesthetic restorations, these values are very close to the limit value of 400 mW/cm^2^ for safe polymerization. After the application of Al_2_O_3_ and laser surface treatments, the amount of transmitted light decreased below 400 mW/cm^2^. The International Organization for Standardization (ISO) recommends an intensity for polymerization lights of 300 mW/cm^2^, and the standard depth-of-polymerization requirement is 1.5 mm. It was stated that *“polymerization lights with an intensity of 300 mW/cm*^*2*^*appear to effectively polymerize most resin-based composite materials when appropriate polymerization times are used* [29]. However polymerization adequate is questionable below the output value of 400 mW/cm^2^ [1].

In this in vitro research, different ceramics, which have different chemical structures like lithium disilicate, were not evaluated for light transmission, and this point may be a limitation for this research. Further studies were required to test the effect of different ceramics and thicknesses on light transmission.

## Conclusisons

Within the limitation of the present study it can be concluded that; the laser and Al_2_O_3_ applications reduced the light transmission of 1.5 mm thickness feldspathic ceramic material below the value of 400 mW/cm^2^ which is critical limit for adequate polymerization.

## Abbreviations

LED, light emitting diode; 3D, three dimesional; SEM, Scanning Electron Microscopy
